# Non-specific amplification of human DNA is a major challenge for 16S rRNA gene sequence analysis

**DOI:** 10.1038/s41598-020-73403-7

**Published:** 2020-10-01

**Authors:** Sidney P. Walker, Maurice Barrett, Glenn Hogan, Yensi Flores Bueso, Marcus J. Claesson, Mark Tangney

**Affiliations:** 1 CancerResearch@UCC, University College Cork, Cork, Ireland; 2 SynBioCentre, University College Cork, Cork, Ireland; 3 APC Microbiome Ireland, University College Cork, Cork, Ireland; 4 School of Microbiology, University College Cork, Cork, Ireland

**Keywords:** DNA sequencing, Bioinformatics

## Abstract

The targeted sequencing of the 16S rRNA gene is one of the most frequently employed techniques in the field of microbial ecology, with the bacterial communities of a wide variety of niches in the human body have been characterised in this way. This is performed by targeting one or more hypervariable (V) regions within the 16S rRNA gene in order to produce an amplicon suitable in size for next generation sequencing. To date, all technical research has focused on the ability of different V regions to accurately resolve the composition of bacterial communities. We present here an underreported artefact associated with 16S rRNA gene sequencing, namely the off-target amplification of human DNA. By analysing 16S rRNA gene sequencing data from a selection of human sites we highlighted samples susceptible to this off-target amplification when using the popular primer pair targeting the V3–V4 region of the gene. The most severely affected sample type identified (breast tumour samples) were then re-analysed using the V1–V2 primer set, showing considerable reduction in off target amplification. Our data indicate that human biopsy samples should preferably be amplified using primers targeting the V1–V2 region. It is shown here that these primers result in on average 80% less human genome aligning reads, allowing for more statistically significant analysis of the bacterial communities residing in these samples.

## Introduction

This communication highlights off-target amplification of human DNA in 16S rRNA gene sequencing, detailing the circumstances necessary for this to occur, and the effects on ensuing research. Such artefacts are not a universal problem, and only occur in samples containing an overwhelming ratio of human to bacterial DNA. This leaves stool samples and skin samples which contain less than 10% and 90% human DNA respectively, unaffected, but can critically impact on analysis of human biopsy samples, where over 97% of the DNA present is of human origin^1^. Given the increased use of human biopsies from a number of body sites in microbiome research^2–5^, this communication serves as a timely and, to our knowledge, unique methodological warning and remedy, particularly as only one mention of this issue can currently be found in the literature^6^.

Currently, comparisons of primer pairs and the hypervariable regions they target in the 16S rRNA gene have focused exclusively on differing levels of taxonomic resolution and specificity^7,8^. The degree to which bacterial resolution is lost to the production human-derived amplicons has, so far, received no attention. This is because workflows for the analysis of 16S rRNA gene sequencing data typically remove reads falling too far from the mean or median sequence length, or if they are not classified taxonomically as originating from bacterial DNA. This is effective in ensuring that the presence of amplified human DNA does not have any impact on downstream analysis. Unaddressed is the fact that in a sequencing experiment yielding a finite amount of data (up to 15 Gb on a typical Miseq run^9^), a significant proportion of these can be wasted due to this off target amplification. This affects sequencing studies in two ways.

- Prospectively: If this loss of data is anticipated, fewer samples can be sequenced on a given sequencing run, adding to the expense which is already prohibitive for smaller labs.
- Retrospectively: If this loss if data is not anticipated, insufficient bacterial reads may be yielded to accurately characterise the samples being sequenced, particularly if attempting to identify the prevalence of rare taxa between different treatment groups.

**Figure 1:**
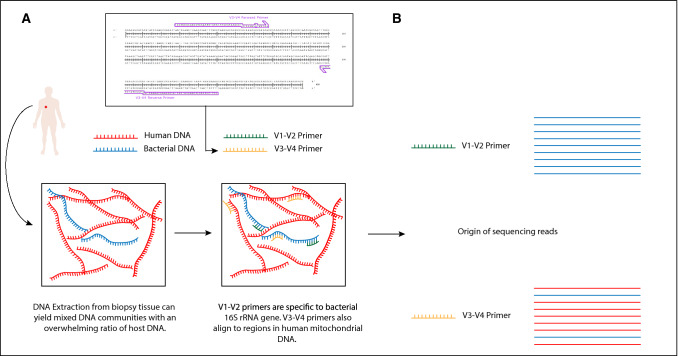
Proposed mechanism for off target amplification of mammalian DNA by V3–V4 primers, as opposed to V1–V2. (**A**) DNA extracted from human biopsies is known to contain large proportions of human DNA. In these circumstances V3–V4 degenerate primers, which also align to region in human mitochondrial DNA as shown can bind and amplify human DNA. There is no such alignment for V1–V2 degenerate primers. (**B**) Off target amplification significantly alters the 16S rRNA gene sequencing profile of a sample.

Here, we show that the most commonly-used primer set for 16S rRNA sequencing, targeting the V3–V4 hypervariable regions, is particularly susceptible to this off-target amplification, while another commonly used primer set, targeting the V1–V2 primer region, shows almost no off-target amplification, as outlined in Fig. 1 below. While this off-target amplification does not appear to affect research using stool or skin swab samples, we would urge all groups carrying out metataxonomic analysis of low microbial biomass human biopsy samples using high throughput sequencing to use the V1–V2 primer set in future.

## Materials/methods

### Sample collection

Breast tissue was collected from women undergoing breast surgery at Cork University Hospital, Cork, Ireland. Breast tumour core-biopsies were aseptically resected using an Achieve 14G Breast Biopsy System (Iskus Health, UT, USA). The specimens were transported in sterile PBS to the lab, where they were flash-frozen and kept at − 80 °C until further processing. DNA from the specimens was purified following the protocol and reagents provided in the Ultra Deep Microbiome Prep (Molzym, GmbH & Co. KG., Bremen, Germany) and eluted in 100 µl of Tris–HCl.

### DNA purification

Samples were processed and DNA purified following the procedures specified in protocols listed in Table 1. In all cases, DNA was eluted in Tris–HCl buffer and stored at − 20 °C until further analysis.

**Table 1:** Samples and corresponding DNA extraction strategy.

Sample	DNA extraction strategy
Breast: tumour and normal	Molzym Ultradeep Microbiome (Molzym, Bremen, Germany)
Oesophageal biopsies	AllPrep DNA/RNA Mini Kit (Qiagen, Hilden, Germany) with modifications^10^
Skin Swab samples	QIAamp UCP Pathogen Mini Kit (Qiagen, Hilden, Germany)
Stool samples	Repeated bead beating method as previously described, with modifications^11, 12^

### 16S rRNA gene sequencing library preparation

Genomic DNA was amplified by PCR with primers targeting the hypervariable V1–V2 region or the V3–V4 region of the 16S rRNA gene. Table 2 details the primers sequences (underlined) included for compatibility with the Illumina 16S Metagenomic Sequencing Protocol (Illumina, CA, USA).

**Table 2:** Primers used for 16S rRNA gene sequencing analysis.

Region	Name	F/R	Sequence
V1–V2^13, 14^	S-D-Bact-0027-b-S-20	F	5′-TCG TCG GCA GCG TCA GAT GTG TAT AAG AGA CAG AGM GTT YGA TYM TGG CTC AG
	S-D-Bact-0338-a-A-18	R	5′-GTC TCG TGG GCT CGG AGA TGT GTA TAA GAG ACA G GCT GCC TCC CGT AGG AGT
V3–V4^15^	S-D-Bact-0341-b-S-17	F	5′ TCG TCG GCA GCG TCA GAT GTG TAT AAG AGA CAG CCT ACG GGN GGC WGC AG
	S-D-Bact-0785-a-A-21	R	5′ GTC TCG TGG GCT CGG AGA TGT GTA TAA GAG ACA G GAC TAC HVG GGT ATC TAA TCC

For Breast Tumour and Normal Adjacent samples, amplification was performed in 50 µl reactions, containing 1X NEBNext High Fidelity 2X PCR Master Mix (NEB, USA), 0.5 µM of each primer, 8 µl template (5–15 ng/µl) and 12 µl nuclease free water. The thermal profile included an initial 98 °C × 30 s denaturation, followed by 25 cycles of denaturation at 98 °C × 10 s, annealing at 55 °C × 30 s for V3–V4 or 62 °C × 30 s for V1–V2 and extension at 72 °C × 30 s. Plus a final extension at 72 °C × 5 min. Amplification was confirmed by running 5 µl of PCR product on a 2% agarose gel, by visualisation of a ≈ 310 bp band for V1–V2 and ≈ 460 bp band for V3–V4.

Faecal microbial genomic DNA was amplified using Phusion High-Fidelity DNA Polymerases (Thermo Scientific, Massachusetts, USA) with the PCR thermocycler protocol as follows: Initiation step of 98 °C for 3 min followed by 25 cycles of 98 °C for 30 s, 55 °C for 60 s, and 72 °C for 20 s, and a final extension step of 72 °C for 5 min.

Oesophageal biopsies and skin swab samples microbial genomic DNA was amplified using MTP Taq DNA Polymerase (Merck KGaA, Darmstadt, Germany) with the PCR thermocycler protocol as follows: Initiation step of 94 °C for 1 min followed by 35 cycles of 94 °C for 60 s, 55 °C for 45 s, and 72 °C for 30 s, and a final extension step of 72 °C for 5 min.

An index PCR was performed to add sample specific DNA barcodes to sample amplicons in accordance with the Illumina 16S Metagenomic Sequencing Protocol (Illumina, California, USA)^16^. Libraries DNA concertation was quantified using a Qubit fluorometer (Invitrogen) using the ‘High Sensitivity’ assay and samples were pooled at a standardised concentration^16^. The pooled library was sequenced on the Illumina MiSeq platform (Illumina, California, USA) utilising 2 × 300 bp chemistry.

### 16S rRNA sequence analysis

The quality of the paired-end sequencing data was visualised using FastQC v (0.11.9), and trimmed using Trimmomatic v (0.39) ensuring a minimum average quality of 25. Reads were then imported into R environment v (3.6.3)^17^ to be resolved into Amplicon Sequence Variants by the DADA2 package v (1.12).

### Contamination control

In all samples a contamination control strategy was implemented in keeping with the RIDE checklist as proposed by Eisenhofer et al.^18^, incorporating aseptic techniques and a variety of negative controls from different stages of the sample-to-sequence data process. Retrospective contamination assessment and removal based on sequencing data from negative controls was also performed following published guidelines^19^.

### Retrospective bioinformatics based removal of human amplicons

Sequencing reads aligning to the human genome (*GRCh38*) within the fasta file generated by DADA2 were identified using bowtie2^20^. To confirm reads mapped to the human genome were not erroneously aligned bacterial reads, all human aligning reads were classified with Mothur^21^, using the RDP database v (11.4) as a reference.

### Statistical analysis and data visualisation

All statistical analysis was carried out in the R environment, using the following libraries: Phyloseq v (1.30), Vegan v (2.5.6), ggplot2 v (3.3.0), reshape2 v (1.4.3).

### Ethical approval

All procedures in this study were performed in accordance to national ethical guidelines, following ethical approval from the University College Cork Clinical Research Committee.

### Informed consent

Patients provided written informed consent for sample collection and subsequent analyses.

## Results and discussion

**Figure 2:**
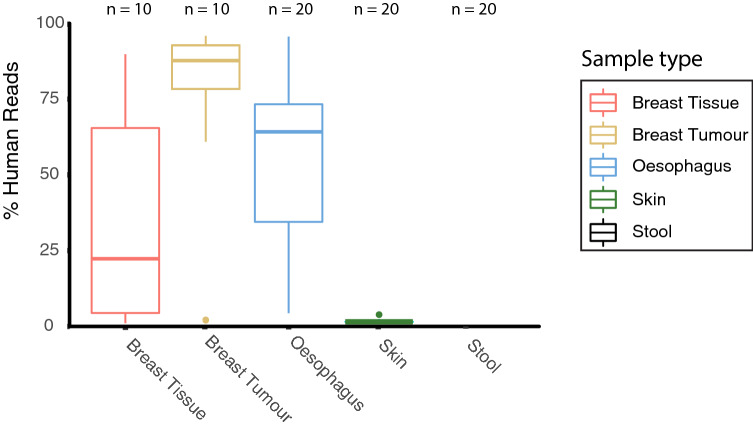
The scale of the problem of off-target amplification. % of sequencing reads produced by Miseq 2 × 300 bp sequencing of amplicons produced by primers targeting the V3–V4 regions shown to align to the human genome.

All three sampled biopsy sites where an overwhelming ratio of host DNA was expected (breast, breast tumour and oesophageal) showed significant off target amplification of human DNA when amplified using the V3–V4 primer set (Fig. 2). This was not seen when sequencing samples with lower levels of human DNA, such as skin swabs and stool samples. An average of 34.1% of all Amplicon Sequence Variants (ASV) detected in normal breast tissue samples were shown to align to the human genome GRCh38 using bowtie2.This included the most prevalent ASV, which was identified further using BLAST as *Homo sapiens haplogroup H8 mitochondrion, complete genome* (Accession no. MN986463.1) with an E-value of 7e − 138 and 100% identity*.* In the breast tumour samples, 77.2% of all ASV’s detected aligned to the human genome, with the most prevalent ASV again being identified as *Homo sapiens haplogroup H8 mitochondrion, complete genome* (Accession no. MN986463.1) with an E-value of 7e − 138 and 100% identity*.* This situation was identical in Oesophageal biopsies, with a 55.6% of ASVs aligning to the human genome (*Homo sapiens haplogroup H8 mitochondrion, complete genome* (Accession no. MN986463.1) with an E-value of 7e − 138 and 100% identity). The skin swab samples showed a much lower level of amplification of human DNA, but these reads aligned to chromosomal DNA, most frequently *Homo sapiens chromosome 17, clone RP11-646F1, complete sequence* and were present in very low levels.

While human contamination is a very common problem in amplification-free shotgun metagenomic sequencing strategies^22^, it is under reported as an issue for 16S rRNA gene sequencing, due to the use of bacteria/archaea specific primers. However, degenerate primers are routinely used for 16S rRNA sequencing^23^. This increases coverage, in terms of the number of 16S rRNA sequences matched by at least one primer, but also allows for off target amplification of non-bacterial DNA. Figure 1A shows that the V3–V4 primers align to a region within the human mitochondrial DNA. We show here that when the ratio of host:bacterial DNA is overwhelming, human mitochondrial DNA can be amplified by primers targeting the 16S rRNA gene region. To ensure the validity of the results, reads identified as aligning to the human genome using Bowtie2 were classified using the Mothur^21^ classifier trained on the RDP database. In all cases the reads identified as aligning to the human genome could not be classified when screened against the RDP database as shown in Table 3 below.

**Table 3:** Summary of Mothur output when classifying reads identified as aligning to the human genome by Bowtie2.

Sample	% reads unclassified at Kingdom Level	% reads unclassified at Phylum level
Oesophageal samples	99.5373235	0.4626765
Normal adjacent samples	98.867576	1.132424
Tumour samples	98.710027	1.289973
Skin samples	99.8588468	0.1411532

**Figure 3:**
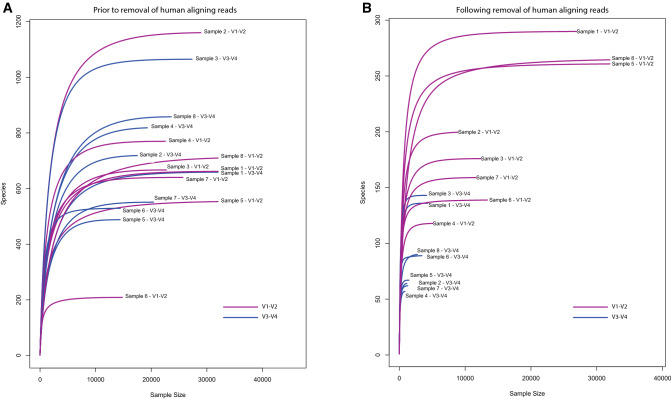
Rarefaction curve generated by plotting observed species vs read depth on a per sample basis. (**A**) Rarefaction curve prior to removal of human genome aligning reads. (**B**) Rarefaction curve following removal of human genome aligning reads.

The most heavily affected sample type in our study (breast tumour tissue) was reanalysed by performing a pairwise comparison of samples amplified with the V3–V4 and V1–V2 primer sets (Fig. 3).

Looking initially at the rarefaction curves produced by the sequencing data corresponding to the previously mentioned paired V1–V2 and V3–V4 primer pair amplified breast tumour sample there is a clear difference between the two groups. This is done by plotting new species against number of reads per sample. Figure 3A below shows that the distribution of samples in this 2D plane appears to be stochastic prior to the removal of human reads. Figure 3B, following removal of human reads, shows clearly that samples amplified with the V1–V2 primer pair consistently yield more observable species, a greater number of reads per sample, and a plateauing of the rarefaction curve which suggests sufficient sampling depth is available for accurate characterisation.

**Figure 4:**
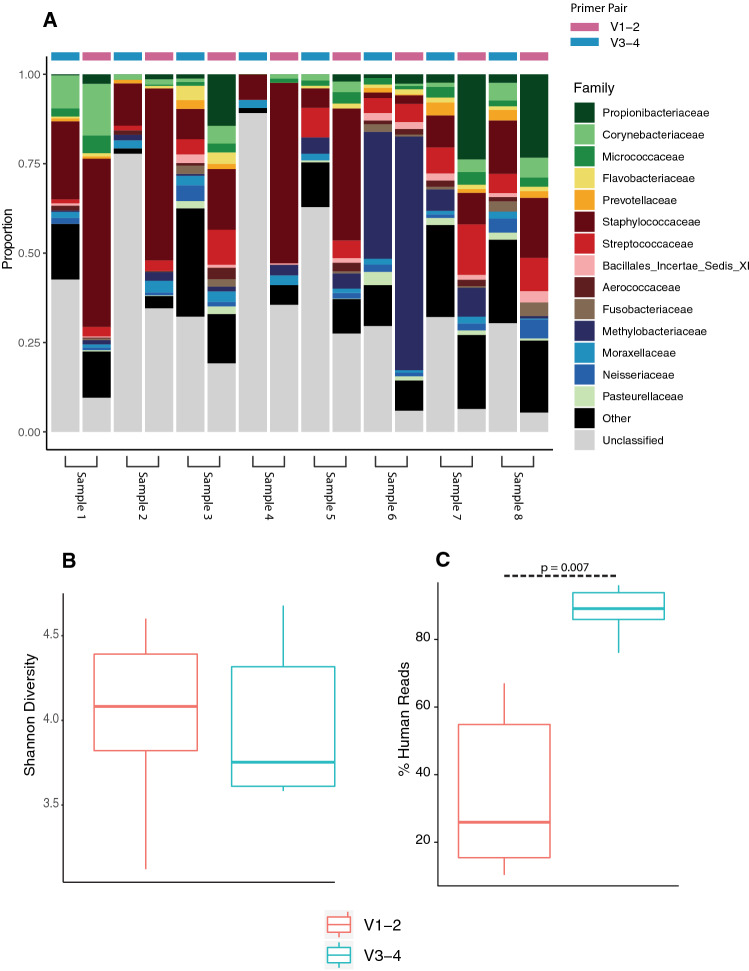
Pairwise comparison of matched samples using primers targeting the V1–V2 and V3–V4 regions of the 16S rRNA gene fragment. (**A**) Sample composition at the family level of paired samples. (**B**) Average Shannon Diversity comparison between samples amplified using V1–V2 primers (red) and V3–V4 primers (blue). (**C**) Percentage of total sequencing reads aligning to human genome. In both (**B**) and (**C**) statistical testing is performed using Wilcoxon signed-rank test.

The community structure in samples amplified with V1–V2 primers was visually similar to those amplified with V3–V4 primers (Fig. 4A) and no bacterial family was found to be significantly elevated using one primer set over the other as per Wilcoxon signed-rank test, once p-values had been corrected for multiple testing using the FDR method (Supplementary Table 1). There was also no significant difference in terms of Shannon diversity (Fig. 4B), indicating choice of primers did not have any adverse effect on the downstream results. Of considerable interest to any groups carrying out low biomass research in the future, is the huge discrepancy in the number of reads yielded once human contamination had been filtered out. As can be seen in Fig. 4C, samples amplified with primers targeting the V1–V2 region have a consistently and significantly higher number of ASVs per sample following the removal of ASV’s aligning to the human genome.

## Future perspectives

Third generation sequencing technologies, such as those produced by Oxford Nanopore Technologies and Pacific BioSiences are now being utilised in 16S rRNA gene sequencing experiments. The Pacific BioSciences SMRT platform has seen the greatest promise in this regard with the implementation of “Circular Consensus Sequencing” in conjunction with denoising algorithms, allowing for the production of long reads of high quality^24^. Earl et al. showed that this new method using degenerate primers targeting the entire 16S rRNA gene, still resulted in off target amplification of the human genome^25^. This study also noted that this off target amplification was related to the ratio of human to bacterial DNA. The human genome must be considered when designing or choosing primers now and in the future.

## Supplementary information

Supplementary Table 1.
