# Supplementary material for: Non-specific amplification of human DNA is a major challenge for 16S rRNA gene sequence analysis
Source: Sci Rep. 2020 Oct 1;10:16356. doi: 10.1038/s41598-020-73403-7 (PMC7529756; doi:10.1038/s41598-020-73403-7)
Supplement: Supplementary file 1 — Supplementary Table 1. [file 41598_2020_73403_MOESM1_ESM.pdf]

**Non-specific amplification of human DNA is a major challenge for 16S rRNA gene sequence analysis**

Sidney P. Walker[1,2]*, Maurice Barrett[3,4]*, Glenn Hogan[1,2], Yensi Flores Bueso[1,2], Marcus J. Claesson[3,4], Mark Tangney[1,2,3]

1) CancerResearch@UCC, University College Cork, Cork, Ireland
2) SynBioCentre, University College Cork, Cork, Ireland
3) APC Microbiome Ireland, University College Cork, Cork, Ireland
4) School of Microbiology, University College Cork, Cork, Ireland

*Both authors contributed equally.

Correspondence:

Mark Tangney PhD MBA, m.tangney@ucc.ie, +353 21 420 5709

# Supplementary Table 1:

| Family | P Value | Adjusted P value |
|---|---|---|
| actinomycetaceae | 0.799846105662473 | 1 |
| actinomycetales_unclassified | 0.932646638965876 | 1 |
| aerococcaceae | 0.799846105662473 | 1 |
| alcanivoracaceae | 1 | 1 |
| bacillaceae_1 | 0.855132140584706 | 1 |
| bacillales_incertae_sedis_x | 1 | 1 |
| bacillales_incertae_sedis_xi | 0.932646638965876 | 1 |
| bacillales_incertae_sedis_xii | 1 | 1 |
| bacillales_unclassified | 0.371093369522698 | 0.866490052 |
| bacilli_unclassified | 1 | 1 |
| bacteria_unclassified | 0.0390625 | 0.355902778 |
| bacteroidales_unclassified | 1 | 1 |
| bacteroidetes_unclassified | 1 | 1 |
| betaproteobacteria_unclassified | 0.371093369522698 | 0.866490052 |
| bifidobacteriaceae | 0.833935414088552 | 1 |
| bradyrhizobiaceae | 1 | 1 |
| brevibacteriaceae | 0.0346105575157073 | 0.355902778 |
| burkholderiaceae | 0.422678074170635 | 0.866490052 |
| burkholderiales_incertae_sedis | 0.789268026134281 | 1 |
| campylobacteraceae | 0.18144920772142 | 0.826601946 |
| candidatus_saccharibacteria_unclassified | 1 | 1 |
| cardiobacteriaceae | 1 | 1 |
| carnobacteriaceae | 0.29450739368011 | 0.866490052 |
| caulobacteraceae | 1 | 1 |
| chitinophagaceae | 1 | 1 |
| clostridiales_incertae_sedis_xi | 0.208412803684149 | 0.854492495 |
| clostridiales_unclassified | 0.787406490666269 | 1 |
| comamonadaceae | 0.41849223344682 | 0.866490052 |
| coriobacteriaceae | 0.371093369522698 | 0.866490052 |
| corynebacteriaceae | 0.0078125 | 0.3203125 |
| dermabacteraceae | 0.18144920772142 | 0.826601946 |
| dermacoccaceae | 1 | 1 |
| dermatophilaceae | 1 | 1 |
| enterobacteriaceae | 0.142213241936393 | 0.826601946 |
| enterococcaceae | 0.422678074170635 | 0.866490052 |
| erysipelotrichaceae | 0.371093369522698 | 0.866490052 |
| eubacteriaceae | 1 | 1 |
| firmicutes_unclassified | 1 | 1 |
| flavobacteriaceae | 0.352542137499713 | 0.866490052 |
| fusobacteriaceae | 0.799846105662473 | 1 |
| fusobacteriales_unclassified | 0.371093369522698 | 0.866490052 |
| gammaproteobacteria_unclassified | 0.371093369522698 | 0.866490052 |
| halomonadaceae | 0.583882420770365 | 1 |

| | | |
|---|---|---|
| intrasporangiaceae | 0.855132140584706 | 1 |
| lachnospiraceae | 0.787406490666269 | 1 |
| lactobacillaceae | 0.18144920772142 | 0.826601946 |
| leptotrichiaceae | 0.401678166469773 | 0.866490052 |
| leuconostocaceae | 1 | 1 |
| listeriaceae | 1 | 1 |
| methylobacteriaceae | 0.015625 | 0.3203125 |
| microbacteriaceae | 0.18144920772142 | 0.826601946 |
| micrococcaceae | 0.0390625 | 0.355902778 |
| moraxellaceae | 0.7421875 | 1 |
| neisseriaceae | 1 | 1 |
| nitrospiraceae | 1 | 1 |
| nocardiaceae | 1 | 1 |
| pasteurellaceae | 0.204893893816379 | 0.854492495 |
| peptostreptococcaceae | 0.371093369522698 | 0.866490052 |
| phyllobacteriaceae | 0.0759269629825577 | 0.622601096 |
| porphyromonadaceae | 0.100348246462291 | 0.740182435 |
| prevotellaceae | 0.0360316862182336 | 0.355902778 |
| promicromonosporaceae | 0.422678074170635 | 0.866490052 |
| propionibacteriaceae | 0.0224942712224497 | 0.355902778 |
| proteobacteria_unclassified | 1 | 1 |
| pseudomonadaceae | 0.371093369522698 | 0.866490052 |
| rhizobiaceae | 1 | 1 |
| rhizobiales_unclassified | 0.18144920772142 | 0.826601946 |
| rhodobacteraceae | 0.280712665268496 | 0.866490052 |
| rhodocyclaceae | 1 | 1 |
| ruminococcaceae | 1 | 1 |
| sphingobacteriaceae | 1 | 1 |
| sphingomonadaceae | 0.280712665268496 | 0.866490052 |
| spirochaetaceae | 1 | 1 |
| sporolactobacillaceae | 1 | 1 |
| sr1_unclassified | 1 | 1 |
| staphylococcaceae | 0.015625 | 0.3203125 |
| streptococcaceae | 0.108319380730004 | 0.740182435 |
| streptophyta | 0.271898710819648 | 0.866490052 |
| thermaceae | 0.371093369522698 | 0.866490052 |
| unknown_unclassified | 0.0078125 | 0.3203125 |
| veillonellaceae | 0.280712665268496 | 0.866490052 |
| xanthomonadaceae | 1 | 1 |

**Supplementary Table 1: Differentially enriched bacterial families between V1-V2 and V3-V4 primer sites.** Significance calculated using Wilcoxon signed-rank test. Once p-values are corrected for multiple testing using FDR method, no families are significantly elevated between primer pairs.
